# Microbiome Analysis of Malacopathogenic Nematodes Suggests No Evidence of a Single Bacterial Symbiont Responsible for Gastropod Mortality

**DOI:** 10.3389/fimmu.2022.878783

**Published:** 2022-04-20

**Authors:** Laura Sheehy, James Cutler, Gareth D. Weedall, Robbie Rae

**Affiliations:** School of Biological and Environmental Sciences, Liverpool John Moores University, Liverpool, United Kingdom

**Keywords:** metagenomics, nematodes, gastropods, symbiosis, biocontrol, bacteria, 16S ribosomal RNA gene analysis

## Abstract

Nematodes and bacteria are prevalent in soil ecosystems, and some have evolved symbiotic relationships. In some cases, symbionts carry out highly specialized functions: a prime example being entomopathogenic nematodes (EPNs), which vector bacteria (*Xenorhabdus* or *Photorhabdus*) into insect hosts, killing them to provide a food source for the nematodes. It is thought that the commercially available malacopathogenic (kills slugs and snails) biocontrol nematode *Phasmarhabditis hermaphrodita* vectors a bacterium (*Moraxella osloensis*) into slugs to kill them. To investigate this further we used a metagenomic approach to profile the bacteria present in the commercial strain of *P. hermaphrodita*, a wild strain of *P. hermaphrodita* and two other *Phasmarhabditis* species (*P. californica* and *P. neopapillosa*), after they had killed their slug host (*Deroceras invadens*). We show that these nematodes do not exclusively associate with one bacterium but a range of species, with members of the phyla Pseudomonadota, Bacillota, Actinobacteriota and Bacteroidota the most prevalent. The commercial strain of *P. hermaphrodita* had the least diverse bacterial community. Furthermore, we found that the bacterium *P. hermaphrodita* has been cultured on for 25 years is not the expected species *M. osloensis* but is *Psychrobacter* spp. and the only strain of the *Phasmarhabditis* species to associate with *Psychrobacter* spp. was the commercial strain of *P. hermaphrodita*. In summary, we found no evidence to show that *P. hermaphrodita* rely exclusively on one bacterium to cause host mortality but found variable and diverse bacterial communities associated with these nematodes in their slug hosts.

## Introduction

**Figure 1:**
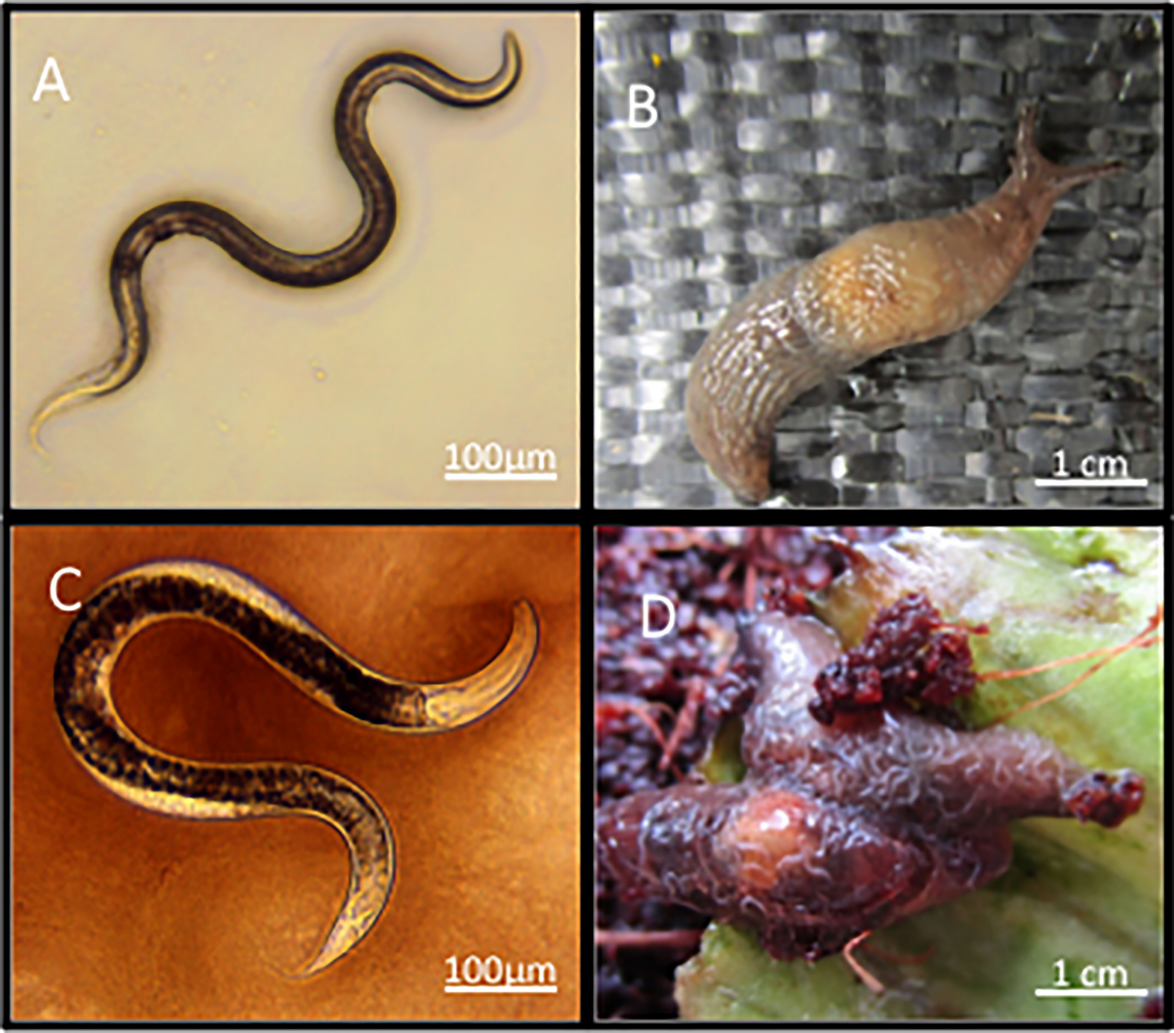
Infective juvenile stage *P. hermaphrodita* **(A)** can infect and kill the susceptible slug *D. invadens* **(B)** and will develop to adults **(C)** and reproduce on the slug cadaver **(D)**.

Nematodes and bacteria are some of the most prolific organisms in soil ecosystems, with numbers of nematodes per gram commonly exceeding 1 million (1) and number of bacterial cells to be approximately 10^10^ (2). Both have evolved relationships ranging from mutualism to parasitism to symbiosis. For example, filarial nematodes (e.g. *Brugia malayi*) rely on intracellular bacteria (*Wolbachia* sp.) for fertility, growth and development (3). Nematodes from the families Stilbonematinae and Desmodoridae carry sulphur-oxidizing bacteria (*Robbea* spp.) on their cuticle, which they rely on for food (4). Entomopathogenic nematodes from the families Steinernematidae and Heterorhabditidae use *Xenorhabdus* and *Photorhabdus* bacteria to kill insect hosts (5). It has been reported a species of malacopathogenic (kills slugs and snails) nematode, *Phasmarhabditis hermaphrodita*, uses a bacterium, *Moraxella osloensis*, to cause death to its terrestrial gastropods, in particular, the grey field slug *Deroceras reticulatum* by producing a lipopolysacharride based endotoxin (6–9). This nematode has been developed as a biological control agent (Nemaslug^®^ available from BASF Agricultural Specialities) to kill slugs on farms and gardens in northern Europe for 25 years (10). The lifecycle of the nematodes begins when they are applied to soil as infective juveniles (**Figure 1A**), which hunt for slugs (**Figure 1B**) and snails in soil by utilising mucus and faecal cues (11). On finding a slug the nematodes enter *via* a pore at the back of the mantle, where they develop to self-fertilising adults (**Figure 1C**) and reproduce (**Figure 1D**) causing a swelling of the mantle area and death in 4-21 days (12, 13). Once this food supply is depleted, new infective juveniles search for new hosts in the soil. *P. hermaphrodita* has been shown to provide significant protection against slug damage in crops such as lettuce, asparagus and Chinese cabbage (10).

The ability of *P. hermaphrodita* to kill terrestrial gastropods and the mechanisms by which they do so are poorly researched. Susceptibility varies with slug species and age, with some larger species such as *Arion ater* and *Arion lusitanicus* resistant to the nematodes (14, 15). Smaller species, such as *D. reticulatum*, are highly susceptible to the nematode (12, 13) and this was initially thought to be due to the introduction and proliferation of the bacterium *M. osloensis* into these hosts *via P. hermaphrodita*. However, the relationship this nematode has with this bacterium is complex and it is not clear whether these nematodes rely on it to kill slugs in nature. Prior to formulation and commercialisation of *P. hermaphrodita* as a biological control agent, Wilson et al. (16, 17) looked for a bacterium that would consistently produce high yields of virulent nematodes and could be used for industrial production. These bacteria were isolated from dying nematode-infected slugs and infective juvenile *P. hermaphrodita.* Of the bacteria tested, *M. osloensis* was chosen, though it must be noted, this was not an ecological study looking at natural isolates used to kill slugs in nature but an exercise to find an isolate to use in commercial production. At the time of writing there is little known about the bacteria *P. hermaphrodita* associates with in nature. Research by Wilson et al. (16, 17) went on to show 24-hour cultures of *M. osloensis* could not kill slugs when injected directly into the slug and cast doubt on the mode of action of the nematodes to vector the bacterium into slugs and cause death. In contrast, it was demonstrated that injection of 60-hour cultures of *M. osloensis* did kill slugs (13), thought to be due to a lipopolysaccharide acting as an endotoxin (7) and using SAGE analysis, genes such as ubiquinone synthetase (*ubiS*) and acyl-coA synthetase (*acs*) were shown to be upregulated and protein-disulfide isomerase (*dscC*) were essential for the virulence process (8). This research led to the idea *M. osloensis* is a symbiont of *P. hermaphrodita*, solely responsible for causing death to slugs. However, contrary to this, other studies have shown that these nematodes can kill slugs without *M. osloensis* (16, 17), the bacterium has never been found in wild *P. hermaphrodita* (18, 19) and it is not retained by infective juvenile nematodes that have killed slugs (20). The idea of this symbiotic relationship is heavily reliant on the paradigm of EPNs, that exclusively rely on one bacterium to kill insect hosts and for development and warrants further investigation.

As well as *P. hermaphrodita* there are another 14 Phasmarhabditis species, yet only a subset have been shown to kill slugs including *P. hermaphrodita, P. papillosa, P. neopapillosa, P. tawfiki, P. safricana, P. bohemica, P. bonaquaense, P. apuliae* and *P. californica* (12, 21–26). There is little information about the pathogenicity of some of these Phasmarhabditis species including *P. neopapillosa*, which is morphologically indistinguishable from closely related *P. hermaphrodita* (22). It has been described as being a lethal parasite of slugs (27), but there are no quantitative data investigating its pathogenic potential or bacterial relationships. Similarly, there is lack of information about the recently described species *P. californica* which since its discovery in the USA (28), has been isolated in New Zealand (29), Canada (30) Ireland (31) and Wales (32). In recent studies *P. californica* (as well as *P. hermaphrodita, P. papillosa* and an undescribed Phasmarhabditis species) isolated from the USA were shown to be lethal to the snail Theba pisana (33) and the slug Deroceras reticulatum (26) under laboratory conditions but there is no information about the pathogencity of *P. californica* strains isolated from the U.K (32). or what bacteria they associate with.

As the role of potential bacterial symbionts in causing mortality to slugs by members of the *Phasmarhabditis* genus is unclear we used 16S ribosomal RNA Metagenomic profiling of *P. hermaphrodita*, *P. californica* and *P. neopapillosa* after killing a slug host (*Deroceras invadens*). Our results are inconsistent with a symbiotic relationship between these nematodes and *M. osloensis* and an exclusive role for the bacterium in causing host mortality, suggesting a model of pathogenicity unlike that of the entomopathogenic nematodes.

## Materials and Methods

### Source of Invertebrates

The commercial strain of *P. hermaphrodita* (DMG0001) (Nemaslug^®^) was supplied by BASF Agricultural Specialities. One wild strain of *P. hermaphrodita* (DMG0010), five wild strains of *P. neopapillosa* (DMG0012, DMG0013, DMG0014, DMG0015 and DMG0016) and three strains of *P. californica* (DMG0017, DMG0018 and DMG0019) were freshly grown to the infective juvenile stage on White traps (34) for 21 days (35). Briefly, White traps consist of placing a 5 cm lid of a Petri dish (lined with Whatman number 1 filter paper) inside a 10 cm Petri dish half filled with water. A 5 mm slice of frozen slug (*Limax flavus* – a suitable food source, 35) is added to the 5 cm Petri dish and 50-100 *Phasmarhabditis* nematodes are added. The White trap is sealed with Parafilm^®^ and left for 21 days (32), the nematodes feed and reproduce on the rotting slug and once the food supply is depleted they develop into infective juveniles and graduate into the surrounding water where they can be harvested for experimentation. Wild strains of *P. hermaphrodita, P. californica* and *P. neopapillosa* were initially isolated from slugs (32) and have been kept in culture at Liverpool John Moores University (LJMU) since 2014.

In order to assess whether these nematodes are pathogenic to terrestrial gastropods we used the common slug host *D. invadens*. It is a non-native pest of U.K. agriculture (36) with a worldwide distribution (36) and has been used to test *Phasmarhabditis* pathogenicity in many studies (37). *D. invadens* were collected from greenhouses at LJMU, stored in non-airtight plastic containers and fed lettuce *ad libitum* for 7 days before infection experiments to ensure they were not infected with nematodes. Slugs collected from this area over the last 8 years have never been found to be infected by *Phasmarhabditis* nematodes (Rae, personal observation).

### Assessing the Pathogenicity of *P. hermaphrodita, P. neopapillosa* and *P. californica* to *D. invadens*

We used a standard bioassay to infect *D. invadens* with *Phasmarhabditis* nematodes (35). Infective stage nematodes (*P. hermaphrodita* DMG0001 and DMG0010), *P. californica* (DMG0017, DMG0018 and DMG0019) or *P. neopapillosa* (DMG0012, DMG0013, DMG0014, DMG0015 and DMG0016) were added in doses of either 500 or 1000 nematodes in 2 mls of water to cotton bungs at the bottom of separate 20 ml universal bottles. Triplicate universal bottles were set up for each nematode strain. Two *D. invadens* were added to each tube and a cotton plug was placed on top and the lid loosely closed. The slugs were exposed for 5 days at 10°C in the dark after which they were placed on a 5 cm Petri dish containing a 3 cm diameter disc of lettuce. Petri dishes were then incubated at 10°C for 9 days. Mortality was recorded every 2–3 days. Ten *D. invadens* were used for each strain and the experiment was repeated three times. A no-nematode control (containing water and no nematodes) and *P. hermaphrodita* DMG0001 (also at a concentration of 500 or 1000 nematodes per tube) were run with each dose of wild *Phasmarhabditis* tested. Each experiment was terminated after 14 days. A Log Rank test was used in OASIS (38) to analyse *D. invadens* survival after exposure to *P. hermaphrodita, P. californica* and *P. neopapillosa* at 0, 500 and 1000 nematodes per ml.

### Microbiome Analysis of *Phasmarhabditis* Nematodes

In order to assess the microbiome of *Phasmarhabditis* nematodes, the same experimental set up was used with the same number of *D. invadens* but we focused on the most pathogenic nematode strains, which were: *P. hermaphrodita* (DMG0010), *P. californica* (DMG0018) and *P. neopapillosa* (DMG0014). We also used the commercial strain of *P. hermaphrodita* (DMG0001). The commercial strain of *P. hermaphrodita* was used to understand 1) the bacterial populations present inside these formulated nematodes after commercial production that were grown solely on *M. osloensis* 2) whether these nematodes would retain *M. osloensis* once they had killed a slug host. A sample (containing approx. 5,000 *P. hermaphrodita*) was taken from a fresh 1 week old package of Nemaslug^®^ (containing *P. hermaphrodita* infective stage nematodes mixed with inert clay), washed with distilled water, quantified, surface sterilized and homogenized using the procedures below. The nematodes were split into three samples were designated “C.DMG0001” and had not killed a slug. We also exposed the nematodes from the same pack of Nemaslug^®^ to *D. invadens* (using the protocols outlined above) and allowed the nematodes to infect, kill and proliferate on the carcass of the dead slugs, and develop to infective stage juveniles. These nematodes then underwent washing, surface sterilisation and homogenisation (as outlined below) and were referred to as “DMG0001”.

Slugs that died during the 14 days of exposure to the nematodes were removed, rinsed with sterile water and placed on individual White traps and left for 21 days. After this time the nematodes developed into infective juveniles nematodes and migrated into the surrounding water. Nematodes were harvested from 10 White traps per *Phasmarhabditis* species (*P. hermaphrodita* DMG0001, *P. hermaphrodita* DMG0010, *P. californica* DMG0018 and *P. neopapillosa* DMG0014) and placed in separate 50 ml Falcon tubes. The nematodes were washed in sterile quarter strength Ringer’s solution three times and then treated with 1% Tween 80 to ensure there were no bacteria present on the nematode cuticle (39). Nematodes from each species were split into 3 samples and concentrated in separate 1.5 ml Eppendorfs and homogenised using individual pestles for 3 minutes. DNA was extracted using a Qiagen Tissue DNA extraction kit and used as the template for all downstream analyses. A DNA extraction negative control was shown to be clear of contamination through PCR and gel electrophoresis.

### 16S rRNA Metagenomic Sequencing of Bacteria Present in *Phasmarhabditis* Nematodes

DNA samples were sent for 16S rRNA Metagenomic sequencing (Novogene). The V4 hypervariable region of the 16S rRNA gene was amplified using the primers 515F (5’-GTGCCAGCMGCCGCGGTAA-3’) and 806R (5’-GGACTACHVGGGTWTCTAAT-3’), all PCR reactions were carried out with Phusion^®^ High-Fidelity PCR Master Mix (New England Biolabs). The libraries were generated with NEBNext^®^ UltraTM DNA Library Prep Kit for Illumina and quantified *via* Qubit and Q-PCR. These libraries were sequenced on an Illumina NovaSeq 6000 platform to generate 2x250 bp paired-end reads.

Paired-end reads were merged using FLASH (V1.2.7) (40). Quality filtering on the raw tags were performed under specific filtering conditions to obtain the high-quality clean tags according to the QIIME (V1.7.0) (41). The tags were compared with the reference database (SILVA database) using UCHIME algorithm (42) to detect chimera sequences. Detected chimera sequences were then removed to obtain Effective Tags. All Effective Tags were processed by UPARSE software (v7.0.1090) (43). Sequences with ≥97% similarity were assigned to the same Operational Taxonomic Units (OTUs).

For each OTUs, QIIME (Version 1.7.0) in Mothur method was performed against the SSUrRNA database of SILVA Database for species annotation at each taxonomic rank (Threshold:0.8~1) (44). MUSCLE (Version 3.8.31) (45) was used to obtain the phylogenetic relationship of all OTUs.

OTUs abundance information was normalized using a standard of sequence number corresponding to the sample with the least sequences. OTUs were analysed for Alpha diversity and Beta diversity to obtain richness and evenness information in samples. Analysis of alpha and beta diversity were all performed on the normalized data and calculated with QIIME (Version 1.7.0)

Principal Component Analysis (PCA) was used to show the differences between samples regarding the structure of microbial community. A One-way Analysis of Variance (ANOVA) with Tukey’s *post hoc* test was used to compare the Shannon index of diversity of microbiomes found in *P. hermaphrodita* (C.DMG0001, DMG0001 and DMG0010), *P. californica* (DMG0018) and *P. neopapillosa* (DMG0014).

### 16S rRNA Amplicon Genotyping of *M. osloensis*

*M. osloensis* used by BASF Agricultural Specialities to grow *P. hermaphrodita* for the last 25 years has never undergone molecular species verification, only identification based on API 20E strips (16, 17). Therefore, we carried out molecular species identification to check whether it was indeed *M. osloensis*. Frozen bacterial cultures of *M. osloensis* from 2002, 2012, 2014 and 2016 were provided by BASF Agricultural Specialities. A single colony of *M. osloensis* was aseptically picked from a nutrient agar streak plate and a 250 ml flask was inoculated with autoclaved nutrient broth. The inoculated culture was left to grow overnight at 28^°^C in a shaking incubator. DNA was isolated from each of the bacterial cultures using a Genejet Genomic DNA purification kit (Thermo fisher™).

For Sanger sequencing, PCR amplification of the hypervariable regions of the 16S rRNA gene was carried out using the primers 27f (5’-AGAGTTTGATCMTGGCTCAG-3’) and 1492r (5’-TACGGYTACCTTGTTACGACTT-3’) (46) and the following conditions: 3 min at 95°C followed by 35 cycles of 15 seconds at 95°C, 30 seconds at 55°C, 1.5 min at 72°C and a final step of 8 mins at 72°C. Amplicons were visualized using agarose gel electrophoresis to ensure that the PCRs had worked correctly; in all cases bands of the correct size were present and no amplification of bacterial DNA could be seen in the negative controls.

Samples underwent Sanger sequencing (Eurofins LightRun Tube Sequencing Service). Consensus sequences were constructed and used to query the NCBI Blastn database.

## Results

### Survival of *D. invadens* Exposed to *P. hermaphrodita, P. californica* and *P. neopapillosa*

**Figure 2:**
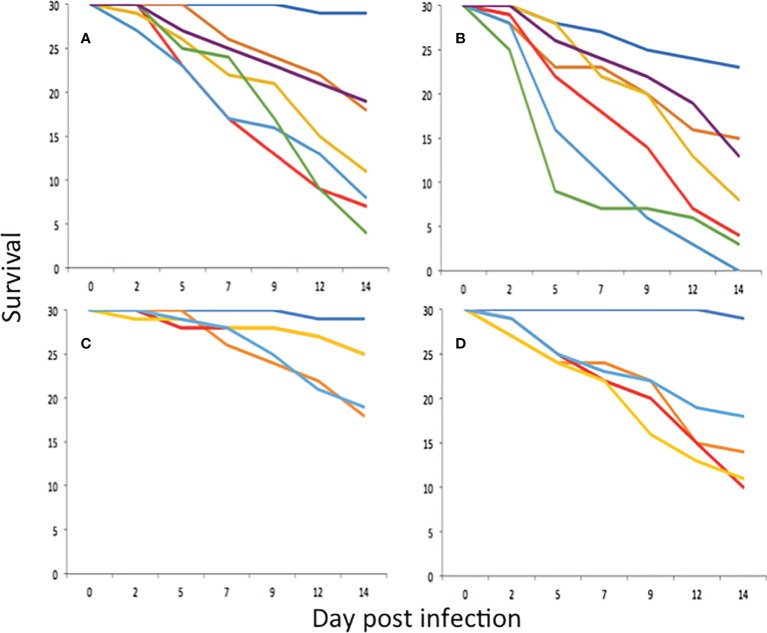
Survival of *D. invadens* (n = 30) exposed to no nematodes (dark blue), *P. hermaphrodita* DMG0001 (orange), *P. neopapillosa* DMG0012 (red), *P. neopapillosa* DMG0013 (yellow), *P. neopapillosa* DMG0014 (light blue), *P. neopapillosa* DMG0015 (green) and *P. neopapillosa* DMG0016 (purple) at dose rates of 500 **(A)** and 1000 **(B)** nematodes per ml. Survival of *D. invadens* (n = 30) exposed to no nematodes (dark blue), *P. hermaphrodita* DMG0001 (orange), *P. californica* DMG0017 (red), *P. californica* DMG0018 (yellow) and *P. californica* DMG0019 (light blue) at dose rates of 500 **(C)** and 1000 **(D)** nematodes per ml.

All *P. neopapillosa* and *P. hermaphrodita* strains caused significant mortality to *D. invadens* compared to the uninfected control at a dose rate of 500 nematodes per ml (P<0.05) (**Figure 2A**) and 1000 nematodes per ml (P<0.05) (**Figure 2B**). At a dose of 500 nematodes per ml, *P. californica* (DMG0019) caused significant mortality to *D. invadens* compared to the uninfected control after 14 days (P<0.05), whereas *P. californica* (DMG0017 and DMG0018) did not (P>0.05) (**Figure 2C**). However, when applied at 1000 nematodes per ml *P. californica* (DMG0017, DMG0018 and DMG0019) caused significant mortality to *D. invadens* compared to the untreated control after 14 days (**Figure 2D**) (P<0.001).

### Bacterial Communities Associated With *Phasmarhabditis* Nematodes After Killing of the Slug Host

**Figure 3:**
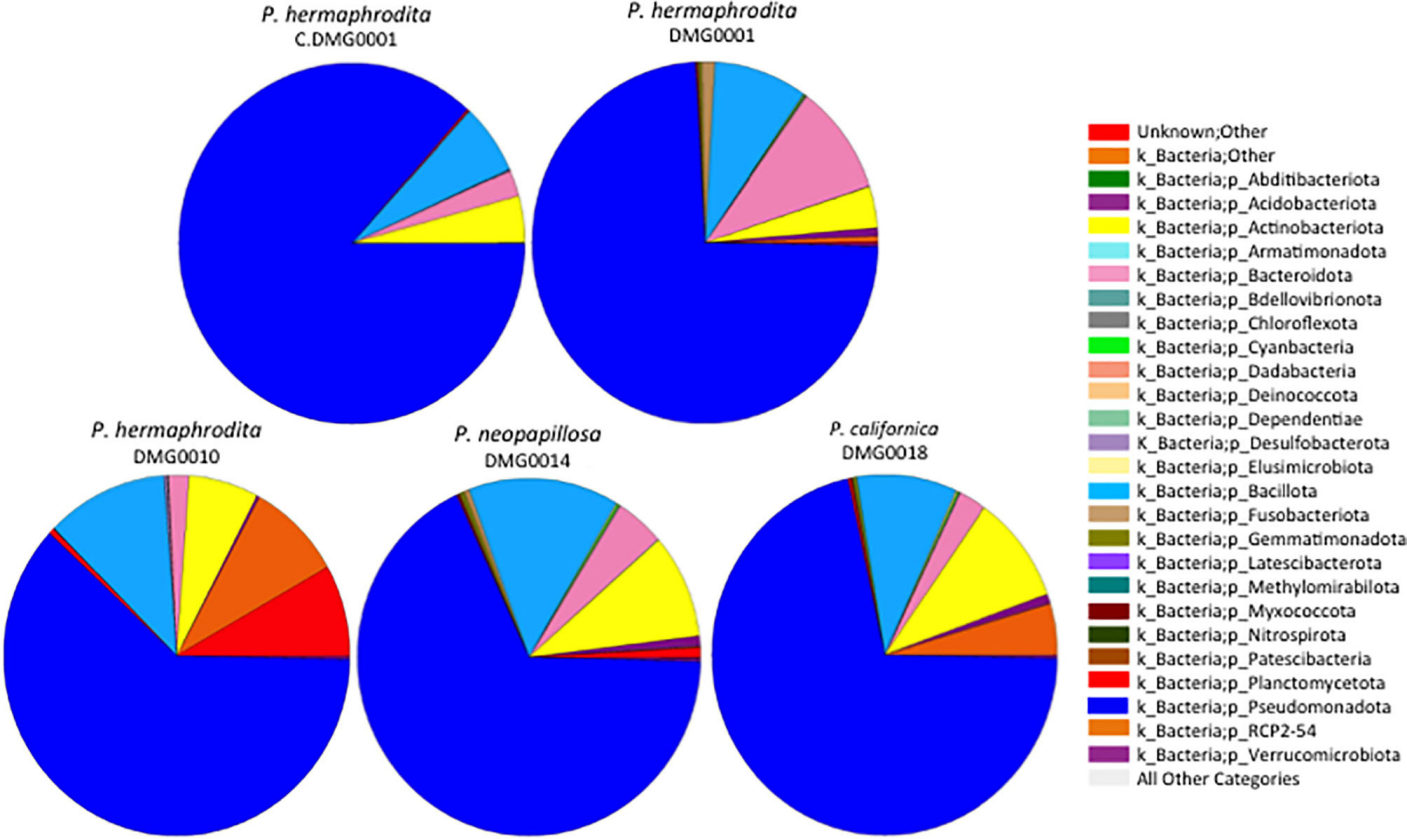
The diversity of bacteria malacopathogenic nematodes associate with: *P. hermaphrodita* (C.DMG0001, DMG0001, DMG0010), *P. californica* (DMG0018) and *P. neopapillosa* (DMG0014). Higher diversity is present in samples which killed a slug host (DMG0001, DMG0010, DMG0014 and DMG0018) whilst the control sample C.DMG0001 has the lowest diversity.

**Figure 4:**
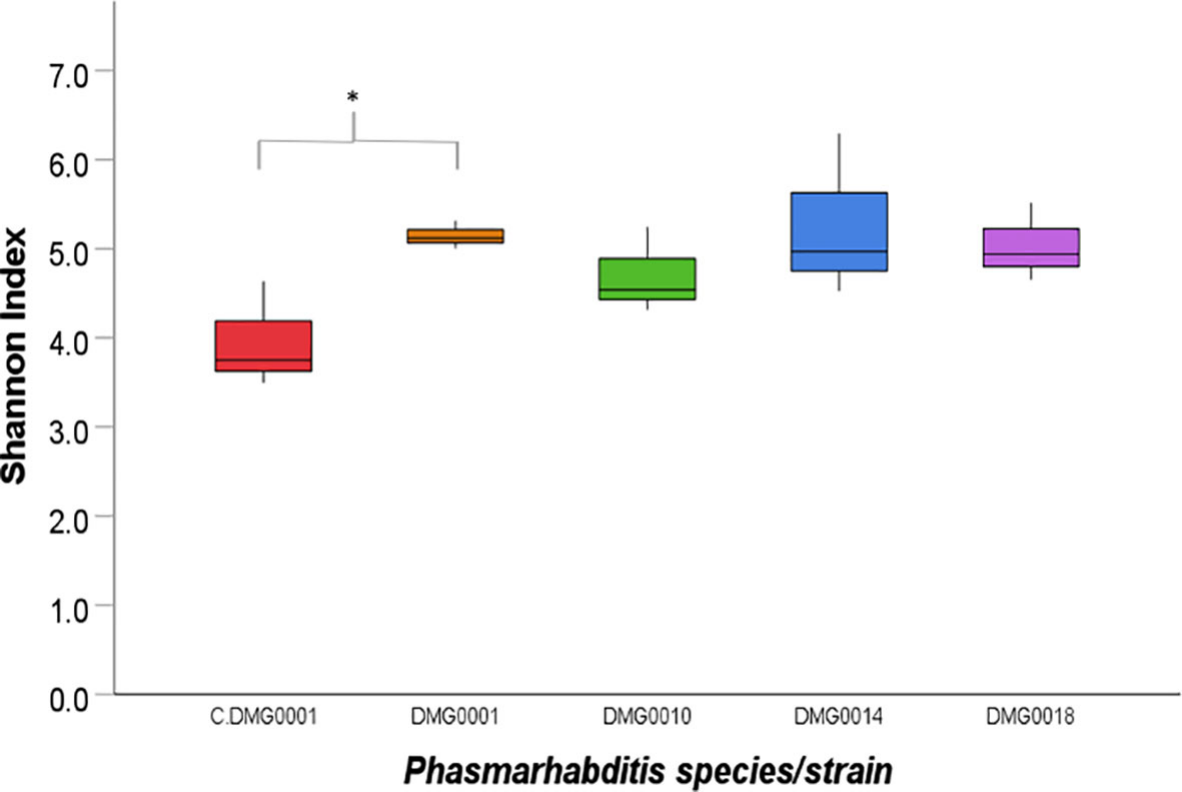
Shannon index of diversity indicates the level of diversity of bacteria found in *P. hermaphrodita* (C.DMG0001, DMG0001, DMG0010), *P. californica* (DMG0018) and *P. neopapillosa* (DMG0014). The lowest diversity was found in *P. hermaphrodita* C.DMG0001. Higher diversity can be seen from nematodes that have killed a slug host: *P. hermaphrodita* (DMG0001 and DMG0010), *P. neopapillosa* (DMG0014) and *P. californica* (DMG0018). * means P < 0.05.

*P. hermaphrodita* (DMG0001 and DMG0010), *P. californica* (DMG0018) and *P. neopapillosa* (DMG0014), which killed *D. invadens* contained a plethora of bacteria from the phyla Pseudomonadota, Bacillota, Actinobacteriota and Bacteroidota (**Figure 3**). This is in stark contrast to *P. hermaphrodita* (C.DMG0001) that did not kill slugs and was taken directly from the pack of Nemaslug^®^, which contained the least diverse set of bacteria (**Figure 4**). In all cases Pseudomonadota was found to be the dominant phylum, with the majority of bacteria belonging to either the class of Alphaproteobacteria and Gammaproteobacteria. Species which killed a slug were found to commonly associate with Gammaproteobacteria, compared to the microbiomes of *P. hermaphrodita* (DMG0010) and *P. hermaphrodita* (C.DMG0001) which had a higher presence of Alphaproteobacteria bacteria. Though it should be noted that *P. hermaphrodita* (DMG0010) had a higher diversity of bacteria belong to Alphaproteobacteria than *P. hermaphrodita* (C.DMG0001).

When the numbers of OTUs were analysed using the Shannon index, the diversity of C.DMG0001 was significantly lower than *P. hermaphrodita* (DMG0001) (P<0.01) but not *P. californica* (DMG0018) and *P. neopapillosa* (DMG0014) (P>0.05) or *P. hermaphrodita* (DMG0010) (P>0.5) (**Figure 4**).

**Figure 5:**
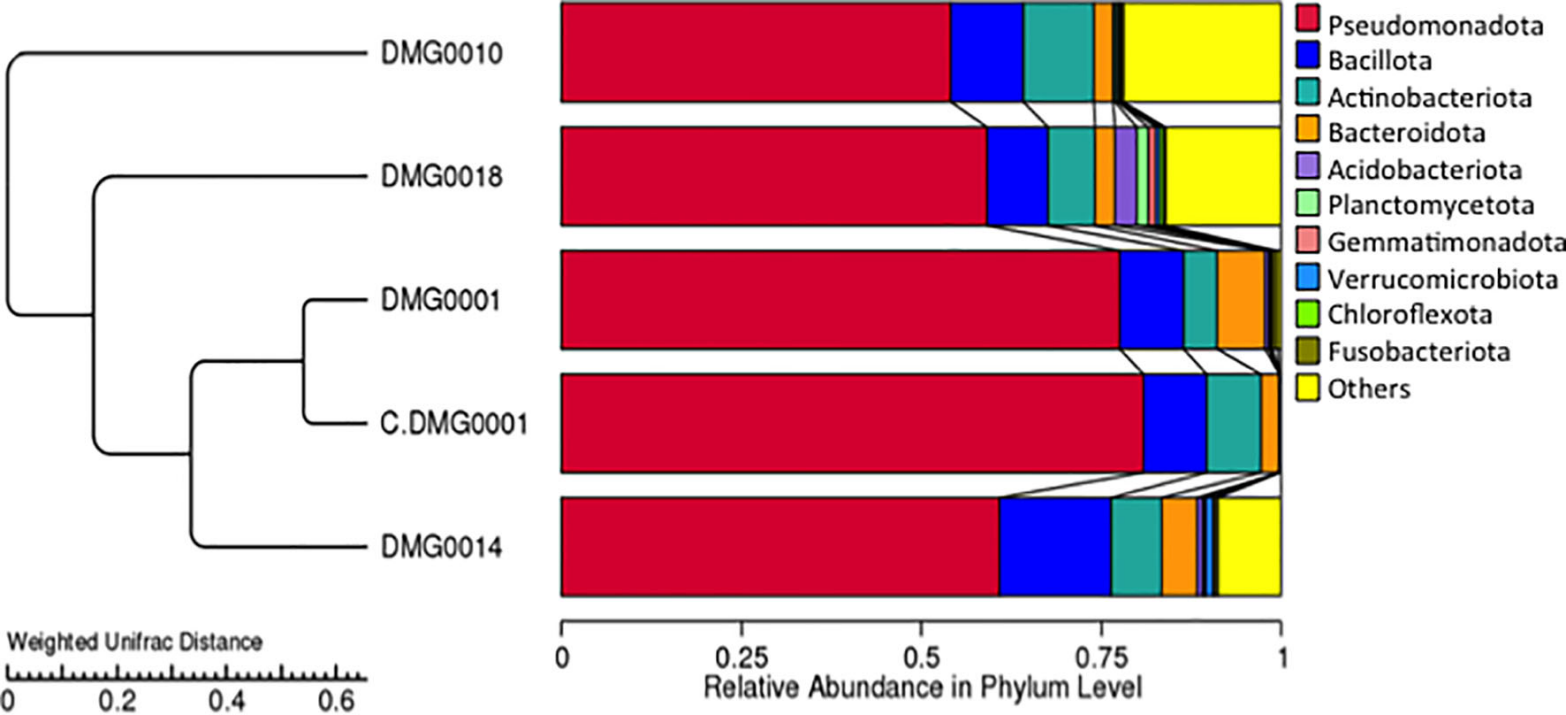
UPGM cluster tree based on Weighted UniFrac distances at the phylum level shows the bacterial microflora is similar between *P. hermaphrodita* C. DMG0001 (which did not kill slugs) and *P. hermaphrodita* DMG0001. The microbiome of *P. neopapillosa* (DMG0014) was closely related to *P. hermaphrodita* (C. DMG0001 and DMG0001), yet *P. californica* (DMG0018) shared little in common with bacteria found in *P. hermaphrodita/P. neopapillosa* clade. The wild strain of *P. hermaphrodita* DMG0010 was the least similar to the other microbiomes.

Although the relative abundance of microbial diversity was lower in *P. hermaphrodita* (C. DMG0001), (taken directly from a packet of Nemaslug^®^ and not exposed to slugs), it was most similar to *P. hermaphrodita* DMG0001 (which had killed slugs) (**Figure 5**). The relative abundance of diversity at the phylum level shows that the microbiome of *P. neopapillosa* (DMG0014) was closely related to *P. hermaphrodita* (C. DMG0001 and DMG0001) than that of *P. californica* (DMG0018), which shared little in common with bacteria found in *P. hermaphrodita.* Interestingly, the microbiomes of the wild strain of *P. hermaphrodita* DMG0010 was the least similar to *P. hermaphrodita* (C. DMG0001) (**Figure 5**). Beta diversity comparison for each sample was completed *via* a Principal Component Analysis (PCA) and showed C.DMG0001 samples were very similar to each other with a lower overall diversity, yet samples which killed a slug host have a much greater diversity of bacteria even within the same *Phasmarhabditis* strain/species (**Supplementary Figure 1**).

**Figure 6:**
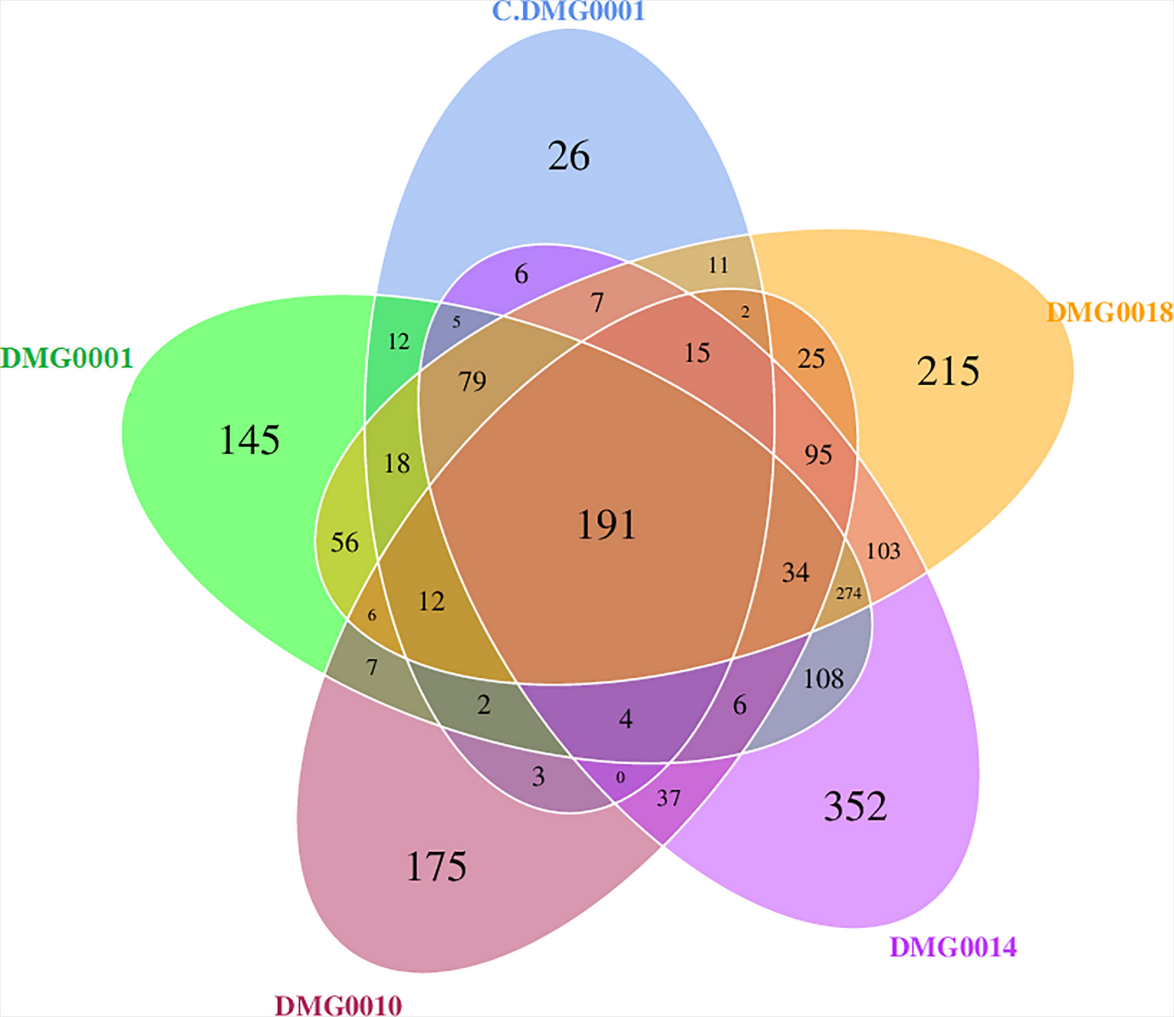
Venn diagram showing malacopathogenic nematodes associate with a core set of bacteria from the phyla Pseudomonadota, Bacillota, Actinobacteriota and Bacteroidota. *P. neopapillosa* (DMG0014) has the high number of unique bacteria (352) which was not found in the other species. *P. hermaphrodita* (C.DMG0001) with did not kill a slug had the lowest unique number of bacteria 26 and the lowest diversity overall.

Each nematode species and strain of *Phasmarhabditis* associated with a core set of microbes, which differed in amount. For example, *P. neopapillosa* (DMG0014) had 352 OTUs and *P. californica* (DMG0018) 215 OTUs whilst *P. hermaphrodita* C.DMG0001 had only 26 unique OTUs and the lowest total number of OTUs of all the samples (393 OTUs). There was a core set of microbes of which all the strains and species share, regardless of whether they killed a slug or not, which totalled to 191 (**Figure 6**). Of these core bacteria 37% belong to the phylum Pseudomonadota, 32% Bacillota, 26% Actinobacteriota and 21% Bacteroidota. The remaining 7% are spread across eight phyla (Chloroflexota, Planctomycetota, Desulfobacterota, Acidobacteriota, Dadabacteria, Deinococcota, Fusobacteriota and Gemmatimonadota).

### The Bacterium Used in Mass Rearing of *P. hermaphrodita* is *Psychrobacter* spp., Not *Moraxella osloensis*

Through 16S rRNA amplicon sequencing it was found that the bacterium *P. hermaphrodita* has been cultured on for 25 years is not *M. osloensis* but *Psychrobacter faecalis* (**Supplementary Figure 2**). The consensus sequences for 16S rRNA gene from the bacterium was compared against the NCBI Nucleotide Collection using BLASTN. Matches with >98% identity were used for species identification. *M. osloensis* only returned an identity match of 92%. This genotyping was repeated for several archived bacterial samples from 2002, 2012, 2014 and 2016 all of which return a result of *P. faecalis >*98% match. A sequence alignment of *M. osloensis* (accession MN758821), *P. faecalis* (accession KC843399) and the consensus sequence (accession ON000493) from the bacteria shows 32 nucleotide differences between *M. osloensis* and the consensus sequence and 20 nucleotide differences between *P. faecalis* and the consensus sequence (**Supplementary Figure 2**). Both *M. osloensis* and *Psychrobacter* belong to the Gammaproteobacteria family of *Moraxellaceae*.

### No Evidence That *Psychrobacter* spp. Is Retained by Nematodes Once They Have Killed Slugs

**Figure 7:**
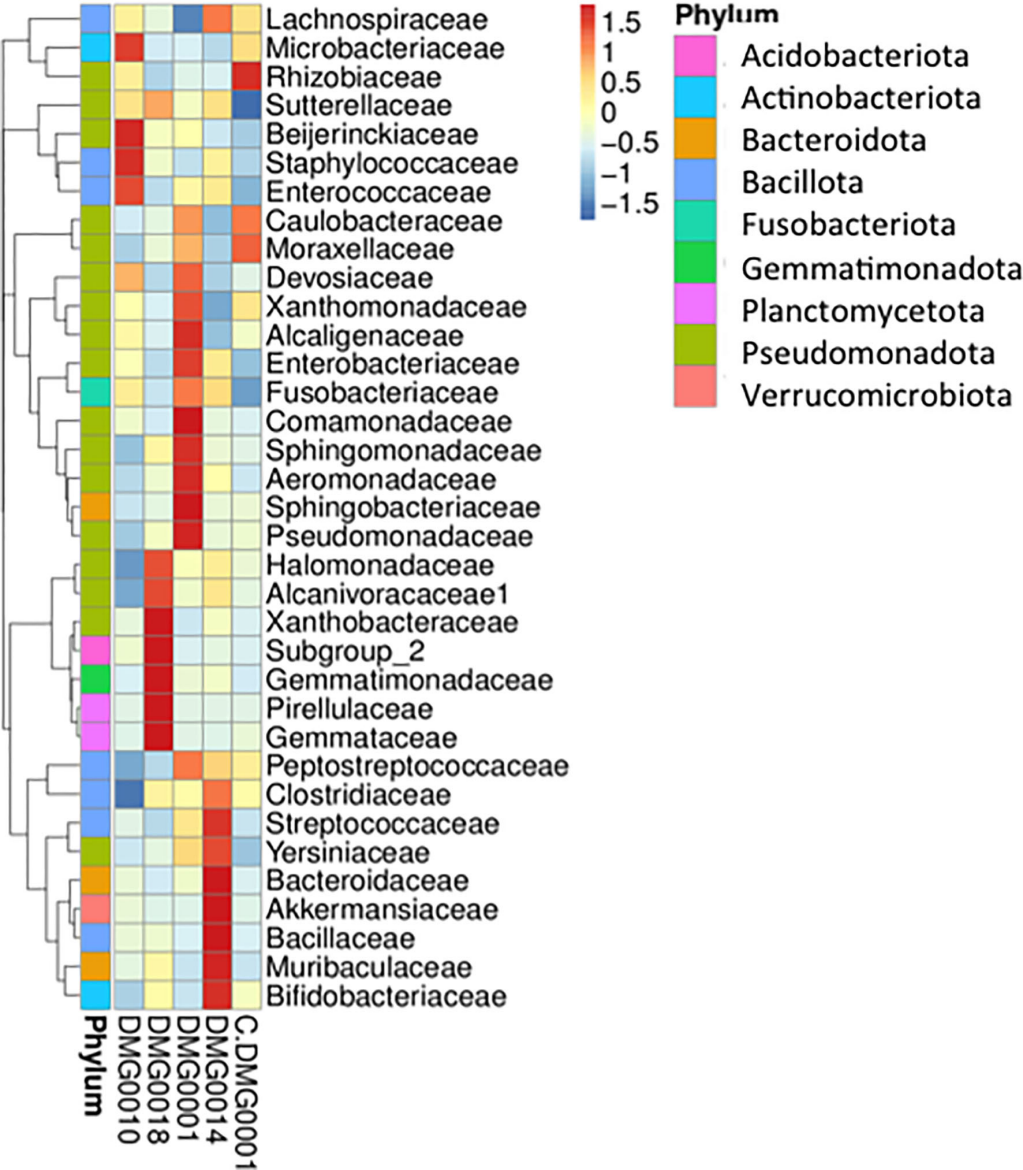
A cluster heatmap shows the presence of certain bacteria vary with different strains and species of *Phasmarhabditis*. There is no evidence of *Psychrobacter* retained by nematodes once they have killed slugs, *Moraxellaceae* (the family which contains *Psychrobacter*) is only abundantly present in *P. hermaphrodita* (C.DMG0001) that did not kill slugs.

The presence of certain bacteria differs with different strains and species of *Phasmarhabditis*. For example, wild *P. hermaphrodita* (DMG0010), which killed *D. invadens*, had an abundance of *Microbacteriaceae, Beijerinckiaceae, Staphylococcaceae* and *Enterococcaceae*, whereas *P. neopapillosa* (DMG0014) had *Bacteroidaceae, Akkermansiaceae, Bacillaceae, Muribaculaceae* and *Bifidobacteriaceae* present and *P. californica* (DMG0018) associated more with *Xanthobacteraceae, Gemmatimonadaceae* and *Pirellulaceae* (**Figure 7**). The commercial strain of *P. hermaphrodita* (C.DMG0001) that did not kill slugs and was taken straight from the pack of Nemaslug^®^ contained *Rhizobiaceae, Moraxellaceae* and *Caulobacteraceae* whereas *P. hermaphrodita* (DMG0001) that did kill slugs had more *Pseudomonadaceae, Sphingobacteriaceae, Areomonadaceae, Sphingomonadaceae* and *Comamonadaceae*. Crucially, it is important to note that *Moraxellaceae* (the family which contains *Psychrobacter*) is only abundant in *P. hermaphrodita* (C.DMG0001) that did not kill slugs.

## Discussion

Until now, the microbiome of *Phasmarhabditis* nematodes was poorly understood. Our results show *M. osloensis* was not retained by *P. neopapillosa*, *P. californica* or *P. hermaphrodita* even though they had killed *D. invadens*, reproduced on its body and developed into new infective stage nematodes. In fact, these wild strains contained a plethora of different bacterial genera and not just one single species (which is what would be expected if these nematodes had the same symbiotic relationship as EPNs). There are several studies that agree with our results and have also failed to find *M. osloensis* in *Phasmarhabditis* nematodes. Recent research (18) used standard microbiological procedures to isolate and culture bacteria from three wild pathogenic *P. hermaphrodita* strains collected from Oregon, U.S.A. Twelve colonies were identified, with no *Moraxella* (or *Psychrobacter*) found. The majority of the bacteria were from the genera *Pseudomonas*, *Sphingobacterium*, *Pseudomonas*, *Acinetobacter*, *Brucella*, *Microbacterium*, *Ochrobactrum* and *Stenotromophonas*. Furthermore, another study (19) showed there was little evidence of vertical transmission of *M. osloensis* to juvenile *Phasmarhabditis* nematodes. Similarly, it was shown using Polymerase Chain Reaction and Denaturing Gel Gradient Electrophoresis (PCR-DGGE) analysis *P. hermaphrodita* infective juveniles, which had killed and reproduced on slugs did not retain *M. osloensis* but were still highly virulent and harboured a large diversity of bacterial species (20).

The retention of one bacterium by members of the *Phasmarhabditis* genus to kill slugs seems unlikely, as these nematodes are dissimilar to EPNs; they are facultative parasites able to reproduce on leaf litter (47), slug faeces (13), dead earthworms (48) and can be reared under lab conditions on many different bacteria (16, 17, 32). Upon death of a slug (due to nematode infection), the microbial communities proliferating on the carcass must be staggering and the possibility of nematodes selectively choosing and ingesting one particular species for future pathogencity seems unlikely. This is in stark contrast to EPNs, which kill insects by introducing a specific bacterium (*Xenorhabdus* for *Steinernema* and *Photorhabdus* for *Heterorhabditis*), which proliferates inside the hard cuticle of an insect and produces antibiotics (49) to outcompete the other bacteria. The cuticle of the insect provides protection against intruding bacteria, and is very different from a decomposing slug open to the environment. Also, EPN associated bacteria (e.g. *Xenorhabdus nematophilus* and *Photorhabdus luminescens*) are exceptionally poor at surviving without their nematode host in soil and water (50), unlike *M. osloensis* which has been found in a range of environments including sinks (51), drains (52) and the ears, noses and throats of hospital outpatients (53). *Psychrobacter* isolates are also commonly found in the environment and from poikilothermic animals (54) with *P. faecalis* found in pigeon faeces (55). There are no reports of *P. faecalis* causing infections or ill health to any species.

For 25 years *P. hermaphrodita* was thought to be grown on *M. osloensis* but our 16S rRNA analysis shows it is more likely *P. faecalis*. *M. osloensis* was part of a collection of bacteria initially isolated from moribund nematode slugs and infective juvenile *P. hermaphrodita* and was identified using the API-20E biochemical test kit (16, 17) and not molecular analysis. Inaccuracies in identification of bacterial species have been reported by researchers using this technique (56). As both species are from the *Moraxellaceae* and are closely related it is plausible incorrect identification took place. The discovery that these nematodes are not a vector of *M. osloensis* is encouraging for the use of *P. hermaphrodita* as concerns have been raised about using a biological agent that could potentially spread an opportunistic human pathogen.

In general, the effect the microbiome has on nematode health is poorly understood. The majority of research has focussed on understanding the effect associated bacteria have on *Caenorhabditis elegans* survival and other life history traits. Three studies (57–59) used a similar approach of sequencing the 16S V4 region and showed *C. elegans* strains isolated from different ecological niches and different geographical regions harboured similar bacteria including Gammaproteobacteria (Enterobacteriaceae, Pseudomonaceae, and Xanthomonodaxeae) and Bacteriodetes (Sphingobacteriaceae, Weeksellaceae, Flavobacteriaceae) and Acetobacteriaceae. When fed these bacteria they had dramatic effects on nematode growth, resistance to biotic and abiotic stressors e.g. resistance to pathogenic bacteria, therefore, proving the native microbiome is crucial for nematode fitness. Though we have not quantified the effect bacteria identified in our study have on *Phasmarhabditis* nematodes previous research has demonstrated different bacterial species can have major effects on *P. hermaphrodita* survival, brood size (32) and virulence (16, 17). It is also interesting to note, that although these different *Phasmarhabditis* species and strains have been isolated from different locations around the U.K., have been kept under lab conditions for different length of time, and were reared in different ways (e.g. on White traps with rotting slugs or commercial production), they still retain a core microbiome consisting of 191 OTUs. Whether these bacteria, or a combination, assist in the pathogenicity of *Phasmarhabditis* nematodes is unknown.

Presumably the bacterial communities present in the intestines of *Phasmarhabditis* nematodes are heavily influenced by the rotting slug. The microbiome of slugs is poorly characterized but one studies found the common black slug (*A. ater*) harboured *Enterobacter*, *Citrobacteri*, *Pseudomonas*, *Escherichia*, *Acinetobacter*, *Pantoea*, *Klebsiella*, *Serratia*, *Erwinia* and *Salmonella* (60). Also the invasive slug *Ambigolimax valentianus* had a core microbiome of *Cirobacteri*, *Delftia*, *Erwinia*, *Arthrobacter*, *Stenotrophominas*, *Pseudomonas*, *Rhodococcus* and *Bacillus*, which was influenced by diet and environment (61). It remains to be discovered whether the microbiome of *Phasmarhabditis* nematodes is strongly influenced by the microbiome of different slug species however, it has recently been reported that when passaged through a slug *C. elegans* retains its native microbiota of *Pseudomonas*, *Chryseobacterium*, *Flavobacterium*, *Pedobacter*, *Lactococcus* and *Pantoea* (62).

In summary, we have shown several *Phasmarhabditis* species are able to parasitise and kill *D. invadens* and the nematodes that proliferate on these hosts contain a wealth of different bacterial phyla and not one bacterial species. Our results support the theory that these facultative parasites do not rely on a single bacterial symbiont, in contrast to EPNs. The precise mechanism these nematodes use to kill slugs remains to be determined, and warrants further attention (32) as they are the only genus in the Nematoda that evolved to do so.

## Data Availability Statement

The data presented in the study are deposited in the ENA repository, accession number PRJEB51844.

## Author Contributions

LS and JC carried out experiments and analysis. RR, GW, and LS wrote the manuscript. RR and JC conceived the study. All authors contributed to the article and approved the submitted version.

## Funding

We are grateful to BASF Agricultural Specialities for funding this research as well as Tom Goddard and Jack Shepherd for discussions.

## Conflict of Interest

The authors declare that the research was conducted in the absence of any commercial or financial relationships that could be construed as a potential conflict of interest.

## Publisher’s Note

All claims expressed in this article are solely those of the authors and do not necessarily represent those of their affiliated organizations, or those of the publisher, the editors and the reviewers. Any product that may be evaluated in this article, or claim that may be made by its manufacturer, is not guaranteed or endorsed by the publisher.

## Supplementary Material

Supplementary Figure 1 Beta diversity comparison for each sample was completed *via* a Principal Component Analysis (PCA). PCA demonstrates that C.DMG0001 samples are very similar to each other with a lower overall diversity, yet all sample which killed a slug show a greater diversity of bacteria.

Supplementary Figure 2 Alignment of *M. osloensis*, *P. faecaelis* and consensus sequence of the bacteria originally thought to be *M. osloensis*. *M. osloensis* 16S sequence (accession number MN758821) and *P. faecaelis* 16S sequence (accession number KM843399) was from NCBI.
