# Supplementary figures and images for: Microbiome Analysis of Malacopathogenic Nematodes Suggests No Evidence of a Single Bacterial Symbiont Responsible for Gastropod Mortality
Source: Front Immunol. 2022 Apr 20;13:878783. doi: 10.3389/fimmu.2022.878783 (PMC9065361; doi:10.3389/fimmu.2022.878783)

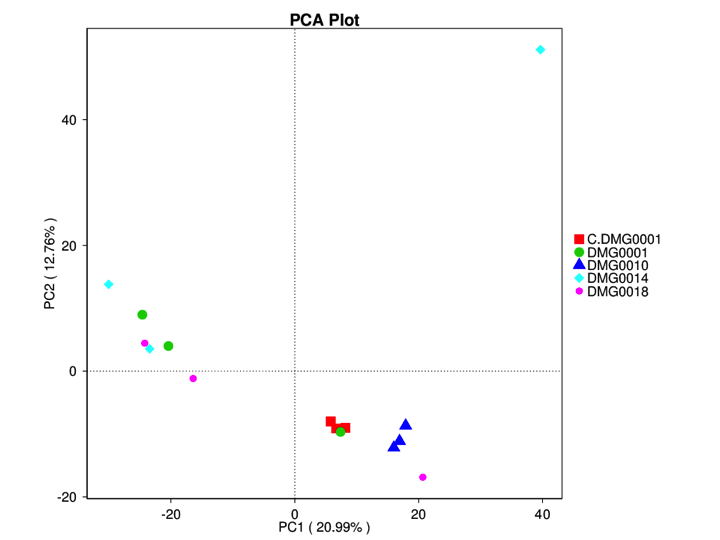

Supplement: Supplementary Figure 1 — Beta diversity comparison for each sample was completed via a Principal Component Analysis (PCA). PCA demonstrates that C.DMG0001 samples are very similar to each other with a lower overall diversity, yet all sample which killed a slug show a greater diversity of bacteria. [file Image_1.tiff]
