# Supplementary material for: Microbiome Analysis of Malacopathogenic Nematodes Suggests No Evidence of a Single Bacterial Symbiont Responsible for Gastropod Mortality
Source: Front Immunol. 2022 Apr 20;13:878783. doi: 10.3389/fimmu.2022.878783 (PMC9065361; doi:10.3389/fimmu.2022.878783)
Supplement: Supplementary Figure 2 — Alignment of M. osloensis, P. faecaelis and consensus sequence of the bacteria originally thought to be M. osloensis. M. osloensis 16S sequence (accession number MN758821) and P. faecaelis 16S sequence (accession number KM843399) was from NCBI. [file DataSheet_1.pdf]

```
Alignment: D:\OneDrive - Liverpool John Moores University\Documents\Laura_PhD_\Publications\Met
Seaview [blocks=10 fontsize=10 A4] on Mon Feb 07 11:15:41 2022

                          1
Cultures_con              GGCGGACGGG TGAGTAATAC TTAGGAATCT ACCTAGTAGT GGGGGATAGC TCGGGGAAAC
Psychrobacter_faecalis    GGCGGACGGG TGAGTAATAC TTAGGAATCT ACCTAGTAGT GGGGGATAGC TCGGGGAAAC
Moraxella_osloensis       GGCGGACGGG TGAGTAACAT TTAGGAATCT ACCTAGTAGT GGGGGATAGC TCGGGGAAAC

                         61
Cultures_con              TCGAATTAAT ACCGCATACG ACCTACGGGA GAAAGGGG-- GCAACTTGTT GCTCTCGCTA
Psychrobacter_faecalis    TCGAATTAAT ACCGCATACG ACCTACGGGA GAAAGGGG-- GCAACTTGTT GCTCTCGCTA
Moraxella_osloensis       TCGAATTAAT ACCGCATACG ACCTACGGGT GAAAGGGGGC GCAA------ GCTCTTGCTA

                        121
Cultures_con              TTAGATGAGC CTAAGTCGGA TTAGCTAGAT GGTGGGGTAA AGGCCTACCA TGGCGACGAT
Psychrobacter_faecalis    TTAGATGAGC CTAAGTCGGA TTAGCTAGAT GGTGGGGTAA AGGCCTACCA TGGCGACGAT
Moraxella_osloensis       TTAGATGAGC CTAAATCAGA TTAGCTAGTT GGTGGGGTAA AGGCCCACCA AGGCGACGAT

                        181
Cultures_con              CTGTAGCTGG TCTGAGAGGA TGATCAGCCA CACCGGGACT GAGACACGGC CCGGACTCCT
Psychrobacter_faecalis    CTGTAGCTGG TCTGAGAGGA TGATCAGCCA CACCGGGACT GAGACACGGC CCGGACTCCT
Moraxella_osloensis       CTGTAACTGG TCTGAGAGGA TGATCAGTCA CACCGGAACT GAGACACGGT CCGGACTCCT

                        241
Cultures_con              ACGGGAGGCA GCAGTGGGGA ATATTGGACA ATGGGGGCAA CCCTGATCCA GCCATGCCGC
Psychrobacter_faecalis    ACGGGAGGCA GCAGTGGGGA ATATTGGACA ATGGGGGCAA CCCTGATCCA GCCATGCCGC
Moraxella_osloensis       ACGGGAGGCA GCAGTGGGGA ATATTGGACA ATGGGGGCAA CCCTGATCCA GCCATGCCGC

                        301
Cultures_con              GTGTGTGAAG AAGGCCTTTT GGTTGTAAAG CACTTTAAGC AGTGAAGAAG ACTCCCATGG
Psychrobacter_faecalis    GTGTGTGAAG AAGGCCTTTT GGTTGTAAAG CACTTTAAGC AGTGAAGAAG ACTCC-ATGG
Moraxella_osloensis       GTGTGTGAAG AAGGCCTTTT GGTTGTAAAG CACTTTAAGC AG-GGAGGAG AGGCTAATGG

                        361
Cultures_con              TTTAATACCC ATGGACGATG ACATTAGCTG CAGAATAAGC ACCGGCTAAC TTCTGTGCCA
Psychrobacter_faecalis    -TTAATACCC ATGGACGATG ACATTAGCTG CAGAATAAGC ACCGGCTAAC -TCTGTGCCA
Moraxella_osloensis       -TTAATACCC ATTAGATTAG ACGTTACCTG CAGAATAAGC ACCGGCTAAC -TCTGTGCCA

                        421
Cultures_con              GCAGCCGCGG TAATACAGAG GGTGCAAGCG TTAATCGGAA TTACTGGGCG TAAAGCGAGC
Psychrobacter_faecalis    GCAGCCGCGG TAATACAGAG GGTGCAAGCG TTAATCGGAA TTACTGGGCG TAAAGCGAGC
Moraxella_osloensis       GCAGCCGCGG TAATACAGAG GGTGCGAGCG TTAATCGGAA TTACTGGGCG TAAAGCGAGT

                        481
Cultures_con              GTAGGTGGCT TGATAAGTCA GATGTGAAAG CCCCGGGCTT AACCTGGGAA CGGCATCTGA
Psychrobacter_faecalis    GTAGGTGGCT TGATAAGTCA GATGTGAAAG CCCCGGGCTT AACCTGGGAA CGGCATCTGA
Moraxella_osloensis       GTAGGTGGCT CATTAAGTCA CATGTGAAAT CCCCGGGCTT AACCTGGGAA CTGCATGTGA

                        541
Cultures_con              TACTGTTAG- GCTAGAGTAG GTGAGAGGAA GGTAGAATTC CAGGTGTAGC GGTGAAATGC
Psychrobacter_faecalis    TACTGTTAG- GCTAGAGTAG GTGAGAGGAA GGTAGAATTC CAGGTGTAGC GGTGAAATGC
Moraxella_osloensis       TACTGGTGGT GCTAGAATAT GTGAGAGGGA AGTAGAATTC CAGGTGTAGC GGTGAAATGC

                        601
Cultures_con              GTAGAGATCT GGAGGAATAC CGATGGCGAA GGCAGCCTTC TGGCATCATA CTGACACTGA
Psychrobacter_faecalis    GTAGAGATCT GGAGGAATAC CGATGGCGAA GGCAGCCTTC TGGCATCATA CTGACACTGA
Moraxella_osloensis       GTAGAGATCT GGAGGAATAC CGATGGCGAA GGCAGCTTCC TGGCATAATA TTGACACTGA

                        661
Cultures_con              GGTTCGAAAG CGTGGGTAGC AAACAGGATT AGATACCCTG GTAGTCCACG CCGTAAACGA
Psychrobacter_faecalis    GGTTCGAAAG CGTGGGTAGC AAACAGGATT AGATACCCTG GTAGTCCACG CCGTAAACGA
Moraxella_osloensis       GATTCGAAAG CGTGGGTAGC AAACAGGATT AGATACCCTG GTAGTCCACG CCGTAAACGA

                        721
Cultures_con              TGTCTACTAG TCGTTGGGTC CCTTGAGGAC TTAGTGACGC AGCTAACGCA ATAAGTAGAC
Psychrobacter_faecalis    TGTCTACTAG TCGTTGGGTC CCTTGAGGAC TTAGTGACGC AGCTAACGCA ATAAGTAGAC
Moraxella_osloensis       TGTCTACTAG CCGTTGGGGT CCTTGAGACT TTAGTGGCGC AGTTAACGCG ATAAGTAGAC

                        781
Cultures_con              CGCCTGGGGG AGTACGGCCG CAAGGTTAAA ACTCAAATGA AATTGACGGG GGGCCCGCAC
Psychrobacter_faecalis    CGCCT-GGGG AGTACGGCCG CAAGGTTAAA ACTCAAATG- AATTGAC-GG GGGCCCGCAC
Moraxella_osloensis       CGCCT-GGGG AGTACGGCCG CAAGGTTAAA ACTCAAATG- AATTGAC-GG GGGCCCGCAC

                        841
Cultures_con              AAGCGGTGGA AGCATGTGGT TTAATTCGAT GCAACGCGAA GAAACCTTAC CTGGGTCTTG
Psychrobacter_faecalis    AAGCGGTGG- AGCATGTGGT TTAATTCGAT GCAACGCGAA G-AACCTTAC CT-GGTCTTG
Moraxella_osloensis       AAGCGGTGG- AGCATGTGGT TTAATTCGAT GCAACGCGAA G-AACCTTAC CT-GGTCTTG
```

```
                            901
Cultures_con                ACATATCTAG AAATCCTGCA GAGAATGCGG AGTGGCCTTC CGGGGAATTA GAAATACAGG
Psychrobacter_faecalis      ACATATCTAG -AATCCTGCA GAGA-TGCGG GAGTGCCTTC --GGGAATTA -GAATACA-G
Moraxella_osloensis         ACATAGTGAG -AATCCTGCA GAGA-TGCGG GAGTGCCTTC --GGGAATTC -ACATACA-G

                            961
Cultures_con                GTGCTTGCAT GGCTGTCGTC AGCTCGTGTC GTGAGATG-T GGGTTAAGTC CCGCAACGAG
Psychrobacter_faecalis      GTGC-TGCAT GGCTGTCGTC AGCTCGTGTC GTGAGATGTT GGGTTAAGTC CCGCAACGAG
Moraxella_osloensis         GTGC-TGCAT GGCTGTCGTC AGCTCGTGTC GTGAGATGTT GGGTTAAGTC CCGCAACGAG

                           1021
Cultures_con                CGCAACCCTT GTCCTTAGTT ACCAGCGGTT TTGGCCGGGA ACTCTAAGGA TACTGCCAGT
Psychrobacter_faecalis      CGCAACCCTT GTCCTTAGTT ACCAGCGGTT T-GGCCGGGA ACTCTAAGGA TACTGCCAGT
Moraxella_osloensis         CGCAACCCTT TTCCTTATTT GCCAGCGGGT TAAGCCGGGA ACTTTAAGGA TACTGCCAGT

                           1081
Cultures_con                GACAAACTGG AGGGAAAGGC GGGGACGACG TCAAGTCATC ATGGCCCTTA CGACCAGGGC
Psychrobacter_faecalis      GACAAACTGG AGG--AAGGC GGGGACGACG TCAAGTCATC ATGGCCCTTA CGACCAGGGC
Moraxella_osloensis         GACAAACTGG AGG--AAGGC GGGGACGACG TCAAGTCATC ATGGCCCTTA CGACCAGGGC

                           1141
Cultures_con                TACACACGTG CTACAATGGT AGGTACAGAG GGCAGCTACA CAGCGATGTG ATGCGAATCT
Psychrobacter_faecalis      TACACACGTG CTACAATGGT AGGTACAGAG GGCAGCTACA CAGCGATGTG ATGCGAATCT
Moraxella_osloensis         TACACACGTG CTACAATGGT AGGTACAGAG GGTTGCTACA CAGCGATGTG ATGCTAATCT

                           1201
Cultures_con                CAAAAAGCCT ATCGTAGTCC AGATTGGAGT CTGCAACTCG ACTCCATGAA GTAGGAATCG
Psychrobacter_faecalis      CAAAAAGCCT ATCGTAGTCC AGATTGGAGT CTGCAACTCG ACTCCATGAA GTAGGAATCG
Moraxella_osloensis         CAAAAAGCCT ATCGTAGTCC GGATTGGAGT CTGCAACTCG ACTCCATGAA GTCGGAATCG

                           1261
Cultures_con                CTAGTAATCG CGGATCAGAA TGCCGCGGTG AATACGTTCC CGGGCCTTGT ACACACCGCC
Psychrobacter_faecalis      CTAGTAATCG CGGATCAGAA TGCCGCGGTG AATACGTTCC CGGGCCTTGT ACACACCGCC
Moraxella_osloensis         CTAGTAATCG CGGATCAGAA TGCCGCGGTG AATACGTTCC CGGGCCTTGT ACACACCGCC

                           1321
Cultures_con                CGTCACACCA TGGGAGTTGA TTGCACCAGA AGTGGGTAGC CTAAC
Psychrobacter_faecalis      CGTCACACCA TGGGAGTTGA TTGCACCAGA AGTGGGTAGC CTAAC
Moraxella_osloensis         CGTCACACCA TGGGAGTCTA TTGCACCAGA AGTAGGTAGC CTAAC
```
